# Real-World Study to Assess Patterns of Treatment Practices and Clinical Outcomes in Metastatic Colorectal Cancer Patients with *RAS* Wild-Type Left-Sided Tumours in Canada

**DOI:** 10.3390/curroncol30090596

**Published:** 2023-09-06

**Authors:** Devon J. Boyne, Elaine Ngan, Chantelle Carbonell, Rajvi J. Wani, Carlye Cirone Morris, Daniel Jun Martinez, Winson Y. Cheung

**Affiliations:** 1 Department of Oncology, University of Calgary, Calgary, AB T2N 4N2, Canada; chantelle.carbonell@ucalgary.ca (C.C.); winson.cheung@albertahealthservices.ca (W.Y.C.); 2 Amgen Canada Inc., Mississauga, ON L5N 0A4, Canada; engan@amgen.com (E.N.); rwani@amgen.com (R.J.W.); ccirone@amgen.com (C.C.M.); dmarti04@amgen.com (D.J.M.); 3 Department of Community Health Sciences, University of Calgary, Calgary, AB T2N 4N1, Canada

**Keywords:** metastatic colorectal cancer, retrospective cohort, RAS testing, overall survival

## Abstract

Minimal Canadian data are available on the RAS testing rates, treatment patterns, and corresponding overall survival (OS) in metastatic colorectal cancer (mCRC) patients. We conducted a population-based cohort study of left-sided RAS wild-type (WT) mCRC patients diagnosed between 1 January 2014 and 31 December 2019, and who were treated with first-line (1L) chemotherapy plus the epidermal growth factor receptor inhibitor panitumumab, chemotherapy plus bevacizumab, or chemotherapy alone, in Alberta, Canada, using electronic medical records and administrative health system data. Of the 2721 patients identified with left-sided mCRC, 320 patients with RAS WT mCRC were treated with 1L systemic therapy: chemotherapy plus panitumumab (n = 64), chemotherapy plus bevacizumab (n = 52), or chemotherapy alone (n = 204). Only 65% and 39% of the 320 1L-treated patients initiated second- and third-line therapy, respectively. A total of 71% of individuals with treated left-sided mCRC underwent RAS testing. The median OS for mCRC patients with RAS WT left-sided tumours was higher for patients treated with 1L panitumumab plus chemotherapy (34.3 months; 95% CI: 23.8–39.6) than for patients who received 1L chemotherapy alone (30.0 months; 95% CI: 24.9–34.1) or 1L bevacizumab plus chemotherapy (25.6 months; 95% CI: 21.2–35.7). These findings highlight an unmet need in left-sided RAS WT mCRC, with relatively few individuals receiving a biologic agent in combination with chemotherapy in the 1L setting, a high rate of attrition between lines, and a need for increased RAS testing before treatment initiation.

## 1. Introduction

Colorectal cancer is the fourth most commonly diagnosed cancer in Canada [1]. Recent Canadian estimates projected 24,300 new colorectal cancer cases and 9400 deaths due to colorectal cancer in 2022, making it the second leading cause of cancer-related deaths in men and the third leading cause of cancer-related deaths in women [1]. Approximately 22% of patients have metastatic disease at presentation [2]. Over the past several decades, fluoropyrimidines have remained the mainstay therapy for metastatic colorectal cancer (mCRC). In the early and late 2000s, additional cytotoxic agents such as irinotecan and oxaliplatin, plus novel monoclonal antibody therapies such as bevacizumab, as well as epidermal growth factor receptor (EGFR) inhibitors including cetuximab and panitumumab were introduced into the Canadian treatment landscape in the first-line (1L) mCRC setting following Health Canada approval of cetuximab in 2012 and panitumumab in 2015 [3,4,5].

There is increasing recognition of significant heterogeneity with colorectal cancer, driven by specific molecular subtypes that can either predict treatment response or prognosticate for survival [6]. EGFR inhibitors have been demonstrated to be associated with a lack of efficacy in the presence of rat sarcoma virus (RAS) mutations (KRAS and NRAS exons 2/3/4) [7,8,9]. Furthermore, primary tumour location (PTL) has been identified as an important predictor of clinical outcomes [10,11]. Post hoc retrospective analysis of pivotal trials and several meta-analyses have demonstrated overall survival (OS) improvements with the addition of EGFR inhibitors to chemotherapy versus chemotherapy +/− bevacizumab in patients with 1L RAS wild-type (WT) left-sided mCRC [12,13,14,15,16]. As such, clinical and molecular markers including PTL should be taken into consideration to guide treatment decisions related to 1L mCRC, as recommended by clinical practice guidelines [17,18] and several Canadian consensus papers [19,20,21,22,23]. The randomized controlled phase 3 PARADIGM study prospectively demonstrated that panitumumab added to mFOLFOX6 led to a median OS benefit of 3.6 months in the left-sided population over bevacizumab plus mFOLFOX6 [24]. EGFR inhibitors plus doublet chemotherapy were added to the ASCO and ESMO guideline updates in late 2022 as the preferred regimen for RAS WT left-sided mCRC [25,26,27]. Further, recent population-based studies in the United States and Australia suggest that treatment with EGFR inhibitors in addition to 1L chemotherapy is associated with improved OS among RAS WT left-sided mCRC patients when compared with bevacizumab plus 1L chemotherapy [28,29].

Nonetheless, very limited published Canadian data are available on the treatment patterns and corresponding OS in mCRC. Furthermore, data on RAS testing rates and detailed analyses related to lines of therapy and type of targeted therapy are also needed. To address these important evidence gaps and add to the increasing literature on population-level treatment patterns and clinical outcomes, this study aimed to characterize the clinical characteristics and outcomes of mCRC patients with RAS WT left-sided tumours treated with 1L chemotherapy in combination with EGFR inhibitors, chemotherapy in combination with bevacizumab, or chemotherapy alone in Alberta, Canada. The secondary and exploratory objectives of this study included the analysis of treatment patterns and attrition of mCRC patients with RAS WT left-sided tumours treated with 1L chemotherapy in combination with EGFR inhibitors, chemotherapy in combination with bevacizumab, or chemotherapy alone; evaluation of the OS and time to next treatment (TTNT) across different lines of systemic therapy; and a description of the timing of receipt of RAS testing results after diagnosis and the time to treatment initiation after RAS testing results among mCRC patients with RAS WT left-sided tumours, respectively.

## 2. Materials and Methods

### 2.1. Study Design and Data Sources

This study was a retrospective longitudinal cohort study of mCRC patients in Alberta, Canada, using real-world, population-level data (Supplementary Figure S1). This study leveraged the administrative databases and the cancer registry that provide coverage for the entire population of Alberta, Canada. The database included 17 cancer centers (2 tertiary, 4 regional, and 11 community hospitals) for the entire province of Alberta, under a public payer system.

### 2.2. Study Population

The study population included adult residents (≥18 years of age) of Alberta, Canada, diagnosed de novo or recurrent RAS WT left-sided mCRC between 2014 and 2019 and who initiated 1L systemic therapy any time post-diagnosis but prior to 31 July 2020. While there exists data on individuals diagnosed prior to 2014, we chose to restrict this investigation to individuals who were diagnosed in 2014–2019, as 2014 was the time when EGFR inhibitors were introduced into the first-line clinical practice in Alberta. Recurrent mCRC patients represent a meaningful portion of the total mCRC population and were therefore included in the study population. Since information on disease recurrence was not directly available in the administrative data, an algorithm developed in collaboration with a senior medical oncologist who treats mCRC in Alberta was used to identify individuals with recurrent mCRC based on meeting any one of the following criteria: (1) receipt of two or more cycles of chemotherapy consistent with an mCRC diagnosis (i.e., fluorouracil, capecitabine) more than 1 year after the date of initial diagnosis, (2) receipt of radiation therapy more than 1 year after the date of initial diagnosis, (3) evidence of RAS testing at any time. Individuals were followed until 31 December 2020, the date of death, or the last date of contact with the health system, depending on whichever occurred first.

### 2.3. Patient Characteristics, Treatment Patterns, RAS Testing, and Survival

The measures in this study were patient clinical characteristics and outcomes, treatment patterns and attrition, OS and TTNT across different lines of systemic therapy, timing of receipt of RAS testing results after diagnosis, and time to treatment initiation after RAS testing result. Baseline demographics were reported for the study cohort overall and stratified by receipt of systemic therapy. Stage was defined according to the most recent edition of the AJCC TNM available at the time of diagnosis (i.e., 6th, 7th, or 8th Edition). For patients with multiple primary tumours, the tumour with the highest stage or, in the case of multiple primaries of the same stage, the earliest diagnosed tumour was used as the index tumour. Baseline demographics included items such as age, sex, geographic location, and Charlson comorbidity. Charlson comorbidity was defined using a claims-based algorithm that was developed and validated using data at the University of Calgary [30]. Continuous baseline characteristics were reported descriptively with mean, standard deviation (SD), and median. Frequencies and percentages were used to document categorical measures of interest. All cell counts with fewer than 10 patients were suppressed (reported as <10 in tables) due to data privacy regulations.

The timing of receipt of RAS testing results after diagnosis was defined as the timing of RAS results in relation to the date of diagnosis. This was presented as a categorical variable (up to 6 months in 1-month intervals), and continuous variables were reported descriptively with mean, SD, and median. Time from RAS testing result to treatment of systemic therapy was defined as the duration of time taken to obtain the first systemic therapy after testing for RAS status. This was presented as a categorical variable (up to 90 days in 2-week intervals), and continuous variables were reported descriptively with mean, SD, and median. The proportions of patients receiving different types of systemic therapies and the median duration of therapy and interquartile range (IQR) were estimated.

A Sankey diagram was included to illustrate the relative sample sizes and proportions of patients receiving different therapies from 1L to second-line (2L) treatment. FOLFOX and CAPOX were combined into a single group because they are clinically synonymous fluoropyrimidine-based regimens, and the sample size was limited. OS and TTNT were examined as time-to-event endpoints. These outcomes were estimated from the time of initiation of 1L systemic therapy until death from any cause (OS) or initiation of the subsequent line of therapy (TTNT). Individuals were censored at the date of last contact with the health care system or on 31 December 2020, depending on whichever occurred first. For OS and TTNT, patients alive at the end of the observation period or who were lost to follow-up were censored. The median time-to-event and the survival proportion at select time points were quantified using the Kaplan–Meier (KM) method. Standard time-to-event analysis using the KM method and the associated medians with associated 95% CIs; KM proportions at select time points (in 1-year intervals); and the number of subjects with events and those censored were used to summarize the findings for OS and TTNT. OS, TTNT, and subsequent treatment regimen types were also examined from initiation of 2L and third-line (3L) therapy.

### 2.4. Ethics Approval

This study was approved by the Health Research Ethics Board of Alberta Cancer Committee (HREBA.CC-22-0039) on 9 January 2022.

## 3. Results

### 3.1. Patient Characteristics

**Figure 1:**
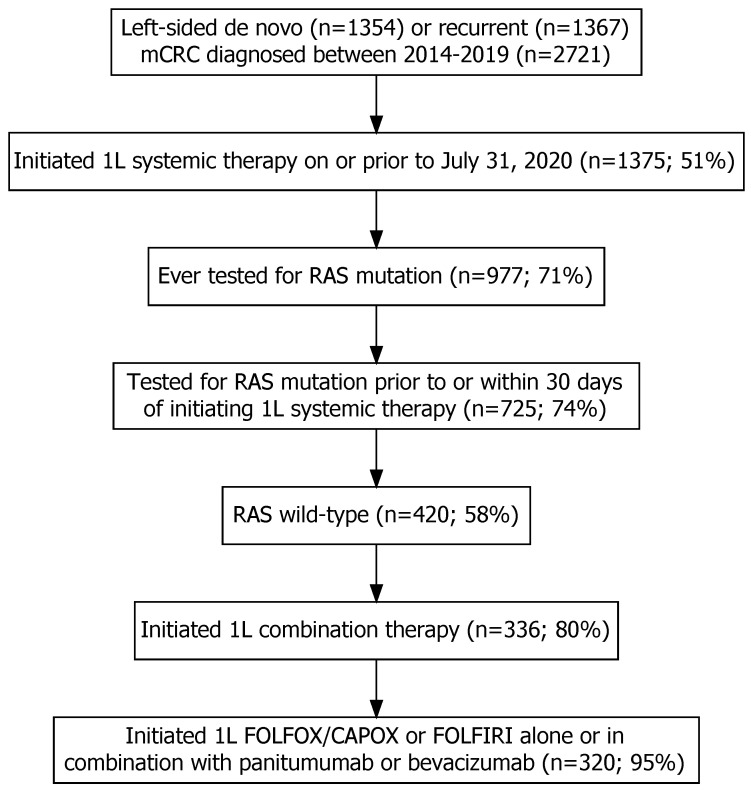
The inclusion and exclusion of individuals with left-sided de novo or recurrent metastatic colorectal cancer (mCRC) into the study after applying relevant eligibility criteria. Note: the percentages were calculated as the number of individuals included in each step divided by the number of individuals included in the previous step multiplied by 100.

This study included 2721 patients with left-sided mCRC (1354 de novo and 1367 recurrent), of which 1375 (51%) were referred and received systemic therapy. Of the 1375 patients, 977 (71%) were tested for RAS mutation, 725 underwent RAS testing prior to or within 30 days of initiating 1L systemic therapy, and 420 were found to have RAS WT status. Among the RAS WT patients, 320 were treated with 1L systemic therapy: chemotherapy in combination with the EGFR inhibitor panitumumab (n = 64), chemotherapy in combination with bevacizumab (n = 52), or chemotherapy alone (n = 204) (Figure 1).

Based on the data analysis, 1L cetuximab was not used in Alberta during the study period. Baseline patient characteristics, stratified according to type of 1L systemic therapy, are presented in Table 1. With respect to differences in baseline characteristics for those who initiated 1L bevacizumab plus chemotherapy, 1L panitumumab plus chemotherapy, versus 1L chemotherapy alone, individuals who received chemotherapy alone tended to be older. In addition, individuals who received 1L panitumumab were more likely to have only one metastatic site, have higher neighbourhood-level income, have 0 Charlson comorbidities, and be diagnosed more recently (Table 1).

### 3.2. Treatment Patterns

**Figure 2:**
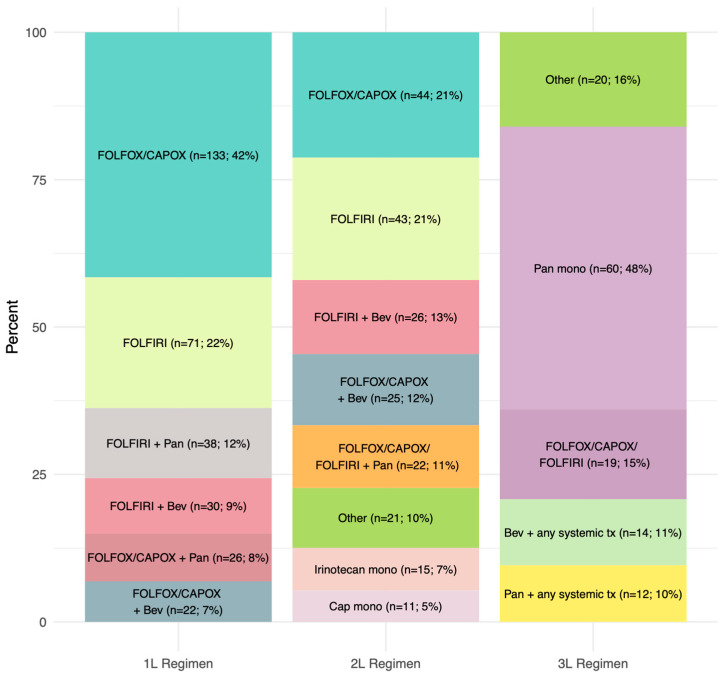
Distribution of systemic therapy according to each line.

Among the 320 RAS WT mCRC patients treated with 1L systemic therapy, 204 (64%) received 1L FOLFOX, CAPOX, or FOLFIRI without biological agents compared to 116 (36%) individuals who received panitumumab or bevacizumab plus chemotherapy (Table 2 and Figure 2). These percentages did not meaningfully change over time. Chemotherapy alone was also the most common form of 2L therapy (65%), whereas panitumumab monotherapy was the primary 3L treatment (48%) (Table 2 and Figure 2). Only 207 (65%) and 125 (39%) of the 320 1L-treated patients initiated 2L and 3L therapy, respectively (Table 2).

**Figure 3:**
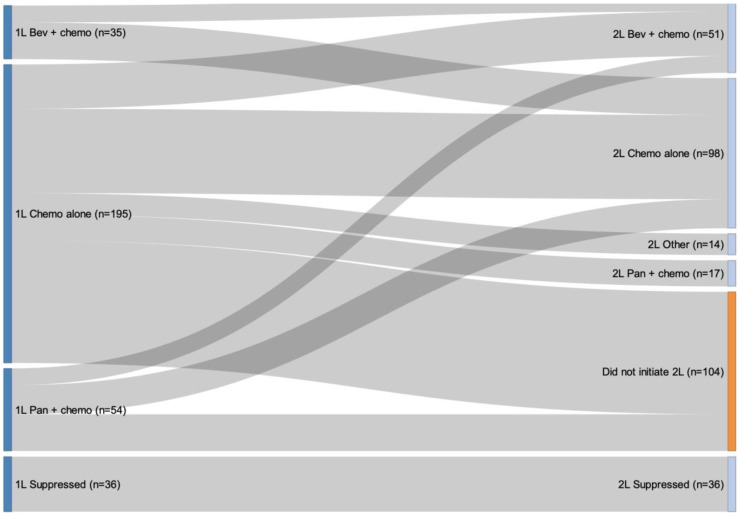
Treatment patterns from first-line to second-line therapy. Note: Of the 320 individuals who initiated 1L treatment, 36 were suppressed in the Sankey diagram analysis due to small cell counts less than 10. The total number of people who were not suppressed was 284. Among those who were not suppressed, there were 180 patients who received 2L treatment.

The sequencing of therapies from 1L to 2L is presented in Figure 3. Of the 320 individuals who initiated 1L treatment, 36 individuals were suppressed in the Sankey diagram analysis due to them being classified in a treatment sequence that had fewer than 10 observations. Of the 284 individuals included in the sequencing analysis, the most common 2L regimen was chemotherapy alone and the majority of individuals who received 1L chemotherapy alone or 1L panitumumab plus chemotherapy did not initiate a 2L therapy (Supplementary Table S1). All patients who received bevacizumab plus chemotherapy for 1L therapy (n = 35) initiated 2L therapy and primarily received chemotherapy alone for 2L therapy (Figure 3). The median duration of 1L systemic therapy was 12.0 months (IQR: 7.4–16.4) for bevacizumab plus chemotherapy, 9.9 months (IQR: 6.3–14.0) for panitumumab plus chemotherapy, and 5.9 months (IQR: 3.0–9.2) for chemotherapy alone.

### 3.3. RAS Testing

The percentage of individuals with treated left-sided recurrent or de novo mCRC who underwent RAS testing was 71% (977/1374). Of these 977 patients, 725 (74%) underwent RAS testing prior to or within 30 days of initiating 1L systemic therapy. Among the 215 de novo mCRC patients included in the analyses, the median time from diagnosis to receipt of RAS testing results was 38 days (IQR: 24–61). The median time from receipt of RAS testing results to the initiation of systemic therapy was 13 days (IQR: 3–26), with a shorter interval among individuals who initiated 1L bevacizumab plus chemotherapy (9.5 days) than those who initiated 1L panitumumab plus chemotherapy (19.5 days).

### 3.4. Overall Survival (OS)

Median OS was 29.4 months (95% CI: 25.6–34.0) from initiation of 1L systemic therapy, 14.4 months (95% CI: 12.8–16.9) from 2L therapy, and 10.2 months (95% CI: 8.8–13.6) from 3L therapy (Table 3). In the 1L setting, the median OS for mCRC patients with RAS WT left-sided tumours was higher for patients who were treated with panitumumab in combination with chemotherapy (34.3 months; 95% CI: 23.8–39.6) compared to patients who received chemotherapy alone (30.0 months; 95% CI: 24.9–34.1) or those who received bevacizumab in combination with chemotherapy (25.6 months; 95% CI: 21.2–35.7) (Table 3).

**Figure 4:**
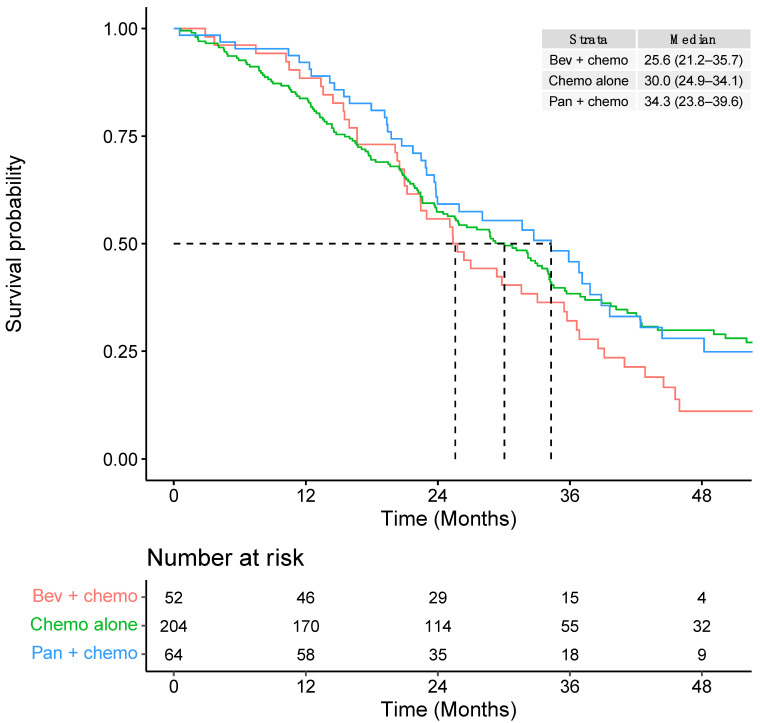
First-line overall survival according to type of chemotherapy regimen within broader systemic therapy groupings.

Patients who received FOLFIRI chemotherapy alone had the shortest median OS of 22.4 months (95% CI: 17.1–28.7) (Supplementary Table S2). Survival curves for each type of 1L systemic therapy (up to 48 months) are presented in Figure 4 and Supplementary Figure S2. Additional survival curves stratified by other patient characteristics are presented in Supplementary Figure S3.

### 3.5. Time to Next Treatment (TTNT)

**Figure 5:**
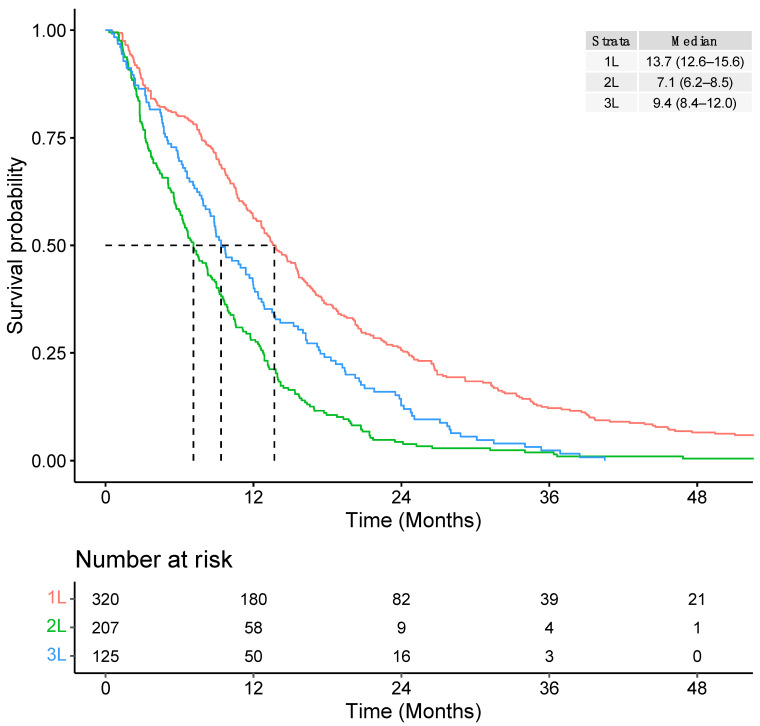
Overall time to next treatment (TTNT) from initiation of each line of therapy.

The median TTNT was 13.7 months (95% CI: 12.6–15.6) from initiation of 1L systemic therapy, 7.1 months (95% CI: 6.2–8.5) from 2L therapy, and 9.4 months (95% CI: 8.4–12.0) from 3L therapy (Figure 5).

## 4. Discussion

This study aimed to describe the clinical characteristics and outcomes of mCRC patients with RAS WT left-sided tumours treated with first-line chemotherapy in combination with EGFR inhibitors, chemotherapy in combination with bevacizumab, or chemotherapy alone in Alberta, Canada, using population-based data. Among the 2721 patients with left-sided mCRC identified in this study, only 1375 (51%) were referred and received systemic therapy. There was a high proportion of individuals who did not initiate systemic therapy in our investigation. Our estimates were based on population-level data which captured information on all cancer patients in the province regardless of referral to a medical oncologist for treatment. The estimated proportion of untreated patients would be much lower in studies that rely exclusively on data from patients who were referred for treatment [31]. Our population-level estimates are aligned with what has been observed in other metastatic cancer sites in the province [31]. Based on our prior work, we expect that the proportion of untreated patients would be ~70% or greater instead of 51% had we restricted the study population to those who were seen by a medical oncologist [31].

Among the 320 patients treated with 1L systemic therapy, 204 (64%) were treated with chemotherapy alone, while only 116 (36%) of patients received a biologic agent (panitumumab or bevacizumab) in combination with chemotherapy. Further, chemotherapy alone was the most common form of 2L therapy (65%). This may be attributable to factors that we were unable to assess because they are not routinely collected in the administrative dataset, including performance score, laboratory measures, and patient or physician preference. There was considerable attrition between lines of therapy, with 65% of individuals initiating 2L therapy and 39% initiating 3L therapy. The progressive decline in the proportion of patients initiating 2L and 3L therapy is consistent with another Canadian study that examined the attrition of patients across lines of systemic treatment for mCRC patients [32]. Out of 200 mCRC patients receiving systemic therapy, Kennecke et al. found that 70% initiated 2L therapy and 30% received 3L therapy [32]. While we could not fully explore the sequencing of therapies due to the limited sample size, there was some suggestion that individuals who received 1L bevacizumab or panitumumab combination therapy were more likely to initiate a second line of therapy compared to those who received 1L chemotherapy alone.

Of the 1374 patients with treated left-sided recurrent or de novo mCRC, 977 (71%) underwent RAS testing. Among these 977 patients, 725 (74%) underwent RAS testing prior to or within 30 days of initiating 1L systemic therapy. According to the Canadian Consensus Practice Guidelines on tumour biomarker testing for mCRC, the minimum biomarker testing required across all Canadian jurisdictions for mCRC patients requires testing for KRAS/NRAS, BRAF, and MMR/MSI prior to the initiation of 1L therapy [33]. The median time from diagnosis to receipt of RAS testing results was 38 days (IQR: 24–61). The Canadian Consensus Practice Guidelines strongly recommend that biomarker testing results should be reported to the medical oncologist by the time of first consultation to inform first-line treatment decisions [33]. They recommend a maximum of 10 working days from sample receipt by the testing laboratory to generation of a summary report, with the report being sent to the referring oncologist within 24 h [33]. Given that these guidelines were published in 2022, we anticipate the proportion of patients tested for additional biomarkers to increase in future cohorts.

Patients treated with 1L bevacizumab in combination with chemotherapy had a shorter median OS (25.6 months; 95% CI: 21.2–35.7) compared to patients who received chemotherapy alone (30.0 months; 95% CI: 24.9–34.1) or patients who were treated with panitumumab in combination with chemotherapy (34.3 months; 95% CI: 23.8–39.6). Patients who received 1L FOLFIRI chemotherapy alone had the shortest median OS of 22.4 months (95% CI: 17.1–28.7) compared to other 1L treatment types. However, this was a descriptive study and comparative efficacy statements cannot be made based on these results since there are several possible explanations for the observed differences in OS, including differences in the study populations, confounding, the timing of treatment initiation, treatment adherence, and resection of metastases.

A strength of this study is that it is among the largest Canadian investigations into RAS WT mCRC due to the availability of province-wide laboratory data. Further, these results have a high degree of generalizability due to the reliance upon population-level data. There is little risk of bias due to differential loss to follow-up due to the use of administrative data to ascertain death and follow-up time. Additionally, treatment data were gathered from electronic medical records, which have a high degree of accuracy and provide coverage for all 17 cancer centres (both academic and community) in the province.

The limitations of this study should also be highlighted. Administrative data algorithms were used to define disease recurrence and lines of therapy which may have resulted in misclassification. However, in the real world, irregular treatment patterns are also not uncommon. Thus, while some of these observations may be attributable to misclassification, several are likely genuine cases of 2L treatment. Due to the use of a recurrence algorithm, we were unable to determine whether individuals had secondary cancers or local recurrences (false positives) and we would have failed to detect recurrences within 1 year of diagnosis (false negatives). The sample size within certain strata was small, which limited our ability to conduct certain analyses, such as the sequencing of therapies. There is a risk of immortal time bias because we included individuals who underwent RAS testing up to 30 days after the date of initiating 1L systemic therapy (i.e., analysis time zero). Since the risk of death within 30 days of initiating 1L systemic therapy was low in this patient population, the risk of immortal time bias would also be low. We did not examine the impact of surgery on survival outcomes according to regimen or line of therapy. Conversion from systemic therapy to surgery may have impacted the outcomes of different regimens. Any statements concerning the comparative efficacy of different systemic therapies should not be made based on these results, as this was a descriptive study that did not control for confounding or other relevant sources of bias. Additional research focused on the emulation of a hypothetical target trial is needed to determine whether any of the observed survival differences were attributable to differences in the real-world efficacy of the therapies examined in this paper [34]. Differences in outcomes between treatments or between this cohort and external trials or real-world cohorts could be due to a number of factors, including differences in the distribution of prognostic factors, levels of treatment adherence, and study eligibility. In Alberta, 1L cetuximab was not used during the study period. As such, these results may not be generalizable to individuals who receive 1L cetuximab therapy.

## 5. Conclusions

In this real-world study of mCRC patients with RAS WT left-sided tumours in Alberta, Canada, our findings highlight an unmet need in left-sided RAS WT mCRC with relatively few individuals receiving panitumumab or bevacizumab in the 1L setting, a need for increased RAS testing prior to treatment initiation, a high rate of attrition between lines, and relatively low survival that declined in subsequent lines. These results highlight the need for careful selection of 1L treatment of mCRC and for additional novel therapeutic options in this patient population.

## Figures and Tables

**Table 1:** Baseline demographics and clinical characteristics of metastatic colorectal cancer patients with RAS wild-type left-sided tumours who initiated systemic therapy, stratified according to type of first-line systemic therapy.

Variable	Overall (n = 320)	Chemotherapy Alone (n = 204)	Bevacizumab Plus Chemotherapy (n = 52)	Panitumumab Plus Chemotherapy (n = 64)
**Demographics**				
Age at index data, years (mean (SD))	56.6 (11.9)	57.8 (12.2)	54.7 (11.7)	54.1 (10.6)
Male (%)	213 (66.6)	38 (73.1)	134 (65.7)	41 (64.1)
Index date 2017-19 (vs. 2014-16) (%) *	182 (56.9)	119 (58.3)	21 (40.4)	42 (65.6)
**Socioeconomic Status**				
Rural residence at initial diagnosis (%)	48 (15.0)	Suppressed	Suppressed	<10 **
Neighbourhood-level annual household income, CAD (mean (SD))	51,247.5 (22,725.7)	50,153.3 (21,588.8)	47,261.6 (17,416.8)	57,973.8 (28,368.5)
Proportion of individuals in neighbourhood with high-school-level education, percent (mean (SD))	80.1 (11.6)	79.5 (11.8)	80.0 (12.6)	81.9 (9.7)
**Comorbidity**				
1+ Charlson comorbidity (%)	99 (30.9)	62 (30.4)	20 (38.5)	17 (26.6)
Diabetes (%)	40 (12.5)	Suppressed	Suppressed	<10 **
Mild liver disease (%)	30 (9.4)	Suppressed	Suppressed	<10 **
**Indicators of Health**				
De novo (vs. recurrent) (%)	215 (67.2)	131 (64.2)	38 (73.1)	46 (71.9)
Colon cancer (vs. rectal) (%)	181 (56.6)	112 (54.9)	29 (55.8)	40 (62.5)
Received systemic therapy at an academic facility (%)	267 (83.4)	172 (84.3)	43 (82.7)	52 (81.2)
Received radiation after index date but prior to initiation of systemic therapy (%)	25 (7.8)	Suppressed	Suppressed	<10 **
Metastatic Sites ***				
2+ metastatic sites (vs. 1)	88 (41.5)	54 (42.2)	19 (50.0)	15 (32.6)
Hepatic met (%)	173 (80.5)	101 (77.1)	35 (92.1)	37 (80.4)
Lymph node met (%)	53 (24.7)	Suppressed	Suppressed	<10 **
Pulmonary met (%)	43 (20.0)	Suppressed	Suppressed	<10 **
Peritoneum met (%)	38 (17.7)	Suppressed	Suppressed	<10 **

* Refers to the date of diagnosis for de novo cases and the date of being flagged as having recurrent disease by the administrative data algorithm for recurrent cases; ** cell counts fewer than 10 were coded as “<10” and the cells for the other treatment groupings were recoded as “suppressed” to prevent the derivation of cell counts under 10; *** due to small cell counts, categories for the number of metastatic sites and index year were collapsed to prevent suppression. With respect to the metastatic site, proportions were estimated among those who presented with de novo metastatic disease, since this information was not available for recurrent cases. Abbreviations: SD = standard deviation.

**Table 2:** Treatment patterns of metastatic colorectal cancer patients with RAS wild-type left-sided tumours according to treatment type and line of therapy.

Variable	Overall (n = 320)
Initiated 1L (%)	320 (100.0)
**1L regimen (%)**	
FOLFOX/CAPOX	133 (41.6)
FOLFIRI	71 (22.2)
FOLFIRI + panitumumab	38 (11.9)
FOLFIRI + bevacizumab	30 (9.4)
FOLFOX/CAPOX + panitumumab	26 (8.1)
FOLFOX/CAPOX + bevacizumab	22 (6.9)
Initiated 2L (%)	207 (64.7)
**2L regimen (%)**	
FOLFOX/CAPOX	44 (21.3)
FOLFIRI	43 (20.8)
FOLFIRI + bevacizumab	26 (12.6)
FOLFOX/CAPOX + bevacizumab	25 (12.1)
FOLFOX/CAPOX/FOLFIRI + panitumumab	22 (10.6)
Other	21 (10.1)
Irinotecan mono	15 (7.2)
Cap mono	11 (5.3)
Initiated 3L (%)	125 (39.0)
**3L regimen (%)**	
Pan mono	60 (48.0)
Other	20 (16.0)
FOLFOX/CAPOX/FOLFIRI	19 (15.2)
Bevacizumab + any systemic therapy	14 (11.2)
Panitumumab + any systemic therapy	12 (9.6)

Abbreviations: 1L = first-line; 2L = second-line; 3L = third-line.

**Table 3:** Median overall survival (months) for metastatic colorectal cancer patients with RAS wild-type left-sided tumours according to treatment type and line of therapy.

Strata	N (%) Initiated	Median Overall Survival, Months (95% CI)
1L overall	320 (100.00)	29.4 (25.6–34.0)
1L chemotherapy alone	204 (63.8)	30.0 (24.9–34.1)
1L bevacizumab + chemotherapy	52 (16.3)	25.6 (21.2–35.7)
1L panitumumab + chemotherapy	64 (20)	34.3 (23.8–39.6)
2L overall	207 (64.7)	14.4 (12.8–16.9)
3L overall	125 (39.0)	10.2 (8.8–13.6)

Abbreviations: CI = confidence interval; 1L = first-line; 2L = second-line; 3L = third-line.

## Data Availability

Individual-level data are not publicly available due to Canadian data privacy laws governing personal health information.

## Supplementary Materials

The following supporting information can be downloaded at https://www.mdpi.com/article/10.3390/curroncol30090596/s1: Figure S1: Study design and research cohort; Figure S2: First-line overall survival according to type of chemotherapy regimen; Figure S3: Overall survival from initiation of 1L according to (A) age at index date; (B) sex; (C) number of Charlson comorbidities; (D) residence at initial diagnosis; (E) time from diagnosis (Dx) to RAS testing (days); and (F) time from RAS testing to 1L (days); Table S1: Sequencing of systemic therapy regimens from 1L to 2L using broader treatment groupings; Table S2: Median overall survival (months) for metastatic colorectal cancer patients with RAS wild-type left-sided tumours treated with first-line therapy, according to treatment type.
