# Supplementary material for: Real-World Study to Assess Patterns of Treatment Practices and Clinical Outcomes in Metastatic Colorectal Cancer Patients with RAS Wild-Type Left-Sided Tumours in Canada
Source: Curr Oncol. 2023 Sep 6;30(9):8220–32. doi: 10.3390/curroncol30090596 (PMC10528146; doi:10.3390/curroncol30090596)
Supplement: Supplementary file 1 [file curroncol-30-00596-s001.zip › curroncol-2553070-supplementary.pdf]

Supplementary Material

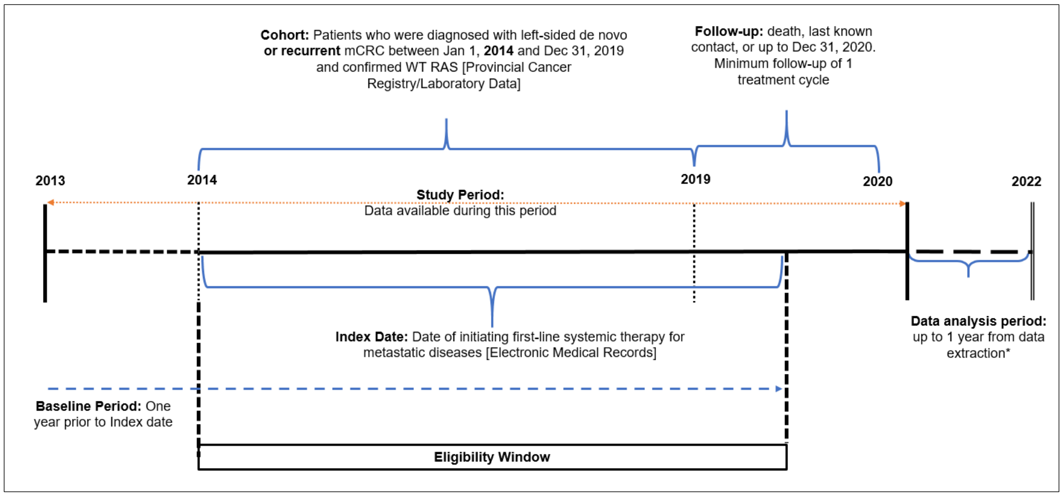

Figure S1. Study design and research cohort.

Baseline Period: For every patient, the baseline period was 1-year prior to index date. The period between January 01, 2014 to December 31, 2019 was used to collect baseline data. In the schema, an example of the baseline period for a patient with an index date of January 01, 2014 is shown. Study Period: Data from as early as January 01, 2013 through to December 31, 2020.

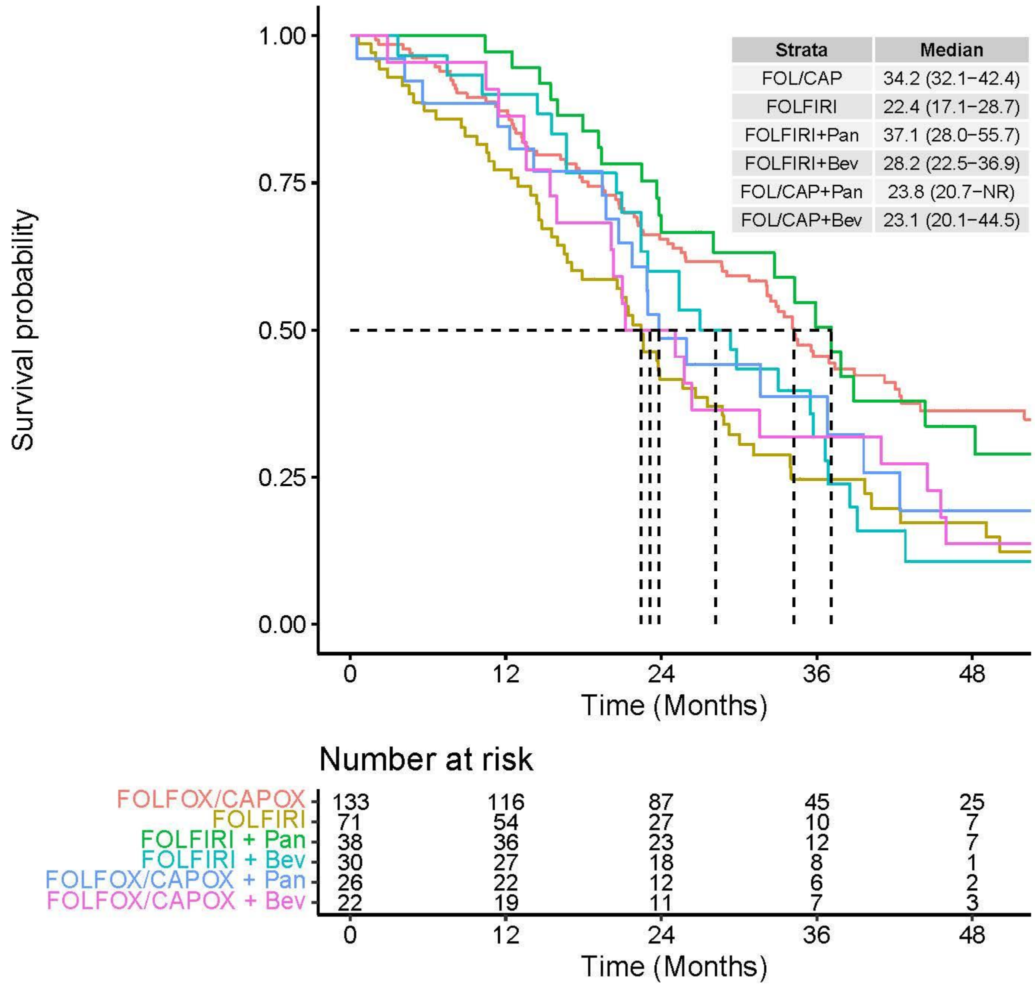

Figure S2. First-line overall survival by type of chemotherapy regimen.

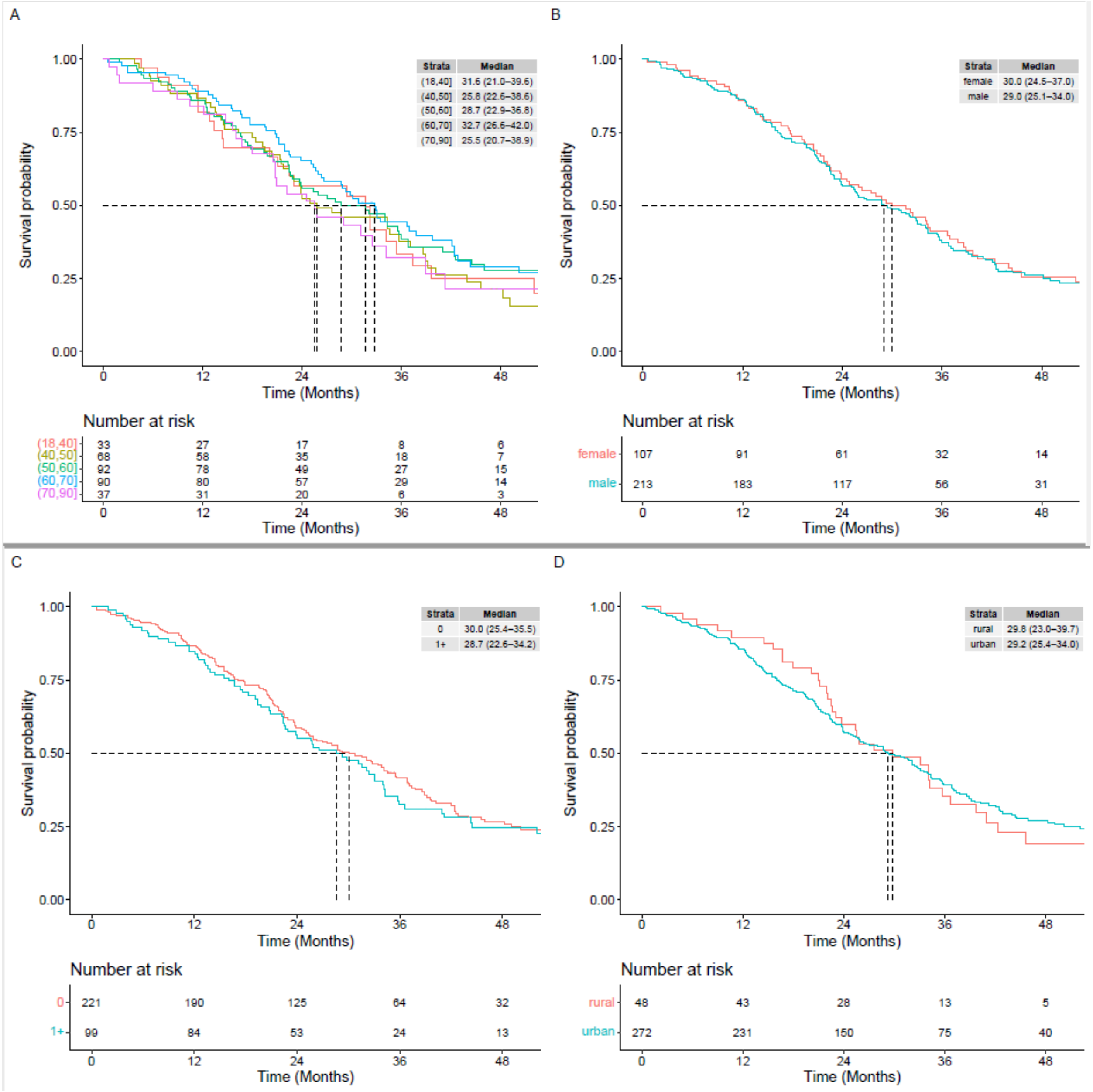
A
B
C
D
Survival probability
Time (Months)
Number at risk
Strata
Median
(18,40] 31.6 (21.0–39.6)
(40,50] 25.8 (22.6–38.6)
(50,60] 28.7 (22.9–36.8)
(60,70] 32.7 (26.6–42.0)
(70,90] 25.5 (20.7–38.9)
female 30.0 (24.5–37.0)
male 29.0 (25.1–34.0)
0 30.0 (25.4–35.5)
1+ 28.7 (22.6–34.2)
rural 29.8 (23.0–39.7)
urban 29.2 (25.4–34.0)
(18,40] 33 27 17 8 6
(40,50] 68 58 35 18 7
(50,60] 92 78 49 27 15
(60,70] 90 80 57 29 14
(70,90] 37 31 20 6 3
female 107 91 61 32 14
male 213 183 117 56 31
0 221 190 125 64 32
1+ 99 84 53 24 13
rural 48 43 28 13 5
urban 272 231 150 75 40

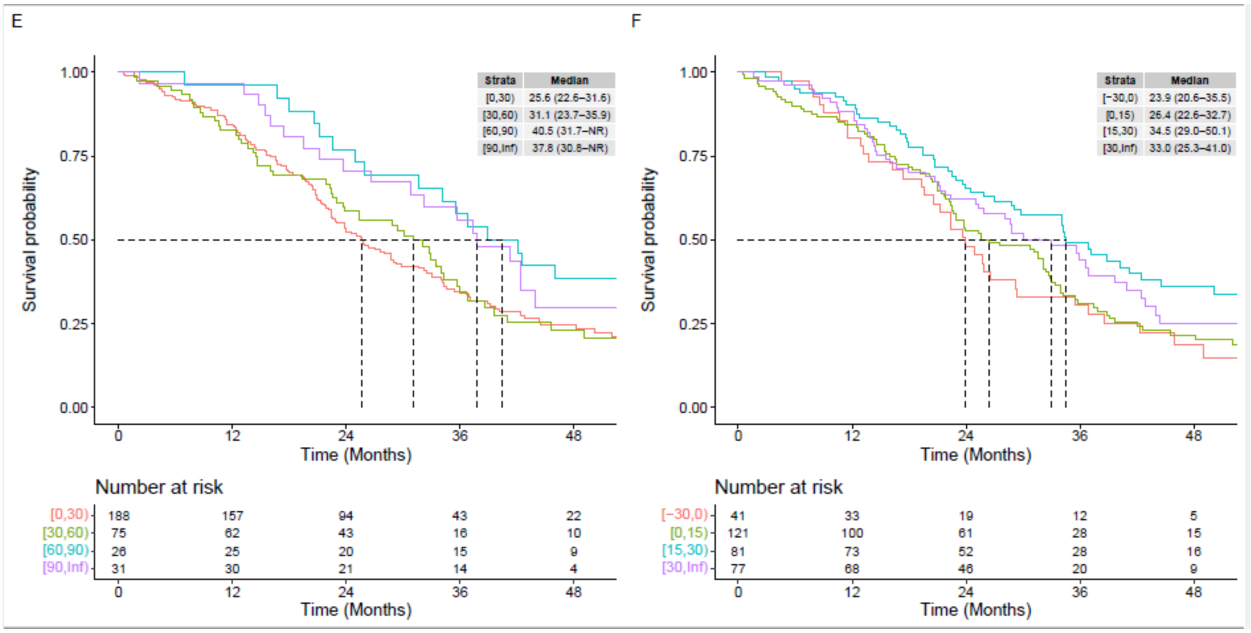

Figure S3 (A-F). Overall survival from initiation of 1L by: (A) Age at Index Date; (B) Sex; (C) Number of Charlson Comorbidities; (D) Residence at Initial Diagnosis; (E) Time from Diagnosis (Dx) to RAS testing (Days); (F) Time from RAS testing to 1L (Days).

Table S1. Sequencing of systemic therapy regimens from 1L to 2L using broader treatment groupings.

1L Regimen	2L Regimen	N	Percent
Bevacizumab + chemotherapy	Bevacizumab + chemotherapy	11	3%
Bevacizumab + chemotherapy	Chemotherapy alone	24	8%
Chemotherapy alone	Bevacizumab + chemotherapy	29	9%
Chemotherapy alone	Chemotherapy alone	55	17%
Chemotherapy alone	Did not initiate 2L	80	25%
Chemotherapy alone	Other	14	4%
Chemotherapy alone	Panitumumab + chemotherapy	17	5%
Panitumumab + chemotherapy	Bevacizumab + chemotherapy	11	3%
Panitumumab + chemotherapy	Chemotherapy alone	19	6%
Panitumumab + chemotherapy	Did not initiate 2L	24	8%
Suppressed	Suppressed	36	11%

Abbreviations: 1L = first-line; 2L = second-line

Table S2. Median overall survival (months) for metastatic colorectal cancer patients with RAS wild-type left-sided tumours treated in first line, by treatment type.

Strata	N (%) initiated	Median overall survival, months (95% CI)
1L Overall	320 (100.00)	29.4 (25.6-34.0)
1L FOLFOX/CAPOX	133 (41.6)	34.2 (32.1-42.4)
1L FOLFIRI	71 (22.2)	22.4 (17.1-28.7)
1L FOLFIRI + bevacizumab	30 (9.4)	28.2 (22.5-36.9)
1L FOLFIRI + panitumumab	38 (11.9)	37.1 (28.0-55.7)
1L FOLFOX/CAPOX + bevacizumab	22 (6.9)	23.1 (20.1-44.5)
1L FOLFOX/CAPOX + panitumumab	26 (8.1)	23.8 (20.7-NA)

Abbreviations: CI = confidence interval; 1L = first-line
